# Diagnosis and treatment of vertigo and dizziness: Interdisciplinary guidance paper for clinical practice

**DOI:** 10.1007/s00106-025-01599-z

**Published:** 2025-06-17

**Authors:** Alexander Andrea Tarnutzer, Hassen Kerkeni, Suzie Diener, Roger Kalla, Claudia Candreia, Renato Piantanida, Raphaël Maire, Antje Welge-Lüssen, Joris Budweg, Andreas Zwergal, Julia Dlugaiczyk

**Affiliations:** 1 Neurology, Cantonal Hospital of Baden, Baden, Switzerland; 2 University of Zurich (UZH), Zurich, Switzerland; 3 Department of Neurology, Bern University Hospital, University of Bern, Bern, Switzerland; 4 Practice Neurology, St. Gallen, Switzerland; 5 Department of Otorhinolaryngology, Head and Neck Surgery, Cantonal Hospital Lucerne, Lucerne, Switzerland; 6 Department of Otolaryngology-Head and Neck Surgery, Ospedale Regionale Di Lugano, EOC, Lugano, Switzerland; 7 Department of Otorhinolaryngology/Head & Neck Surgery, Lausanne University Hospital, Lausanne, Switzerland; 8 Department of Otorhinolaryngology, Head and Neck Surgery, Basel University Hospital, University of Basel, Basel, Switzerland; 9 Hausarztzentrum Bethesda, Basel, Switzerland; 10 Department of Neurology, LMU University Hospital, Munich, Germany; 11 German Center for Vertigo and Balance Disorders (DSGZ), LMU University Hospital, Munich, Germany; 12 Department of Otorhinolaryngology, Head and Neck Surgery, University Hospital Zurich (USZ), Raemistrasse 100, 8091 Zurich, Switzerland; 13 Interdisciplinary Center for Vertigo, Balance and Ocular Motor Disorders, University Hospital Zurich, Zurich, Switzerland

**Keywords:** Ataxia, HINTS, Positional vertigo, Nystagmus, Vestibular syndrome, Ataxie, HINTS, Lagerungsschwindel, Nystagmus, Vestibuläres Syndrom

## Abstract

**Background:**

Vertigo and dizziness belong to the most common leading symptoms in clinical practice. Their differential diagnosis, however, often imposes a challenge.

**Objective:**

This work aims to provide evidence-based and practice-oriented recommendations for diagnosis and treatment of vertigo and dizziness for primary care providers.

**Materials and methods:**

The consensus statement of an interdisciplinary working group following a national survey among Swiss primary care physicians and neurotology specialists (neurologists, otorhinolaryngologists) is presented. The associated literature search in PubMed was conducted up to October 2024.

**Results and conclusion:**

Structured history taking and clinical neurotological examination form the basis for the differential diagnosis of the various acute (AVS), episodic (EVS), and chronic (CVS) vestibular syndromes (AVS: e.g., stroke or acute unilateral vestibulopathy; EVS: e.g., benign paroxysmal positional vertigo [BPPV], Menière’s disease, vestibular migraine, vestibular paroxysmia; CVS: e.g., bilateral vestibulopathy, persistent postural perceptual dizziness). The present paper covers the following topics: i) “red flags” for a potentially dangerous cause in patients with acute vertigo/dizziness/gait and balance disorders; ii) essential clinical neurotological examination steps; iii) diagnostic and therapeutic maneuvers for posterior and lateral canal BPPV; iv) the most important therapeutic strategies for the vestibular syndromes named above; and v) the top 10 recommendations regarding history taking, diagnosis, and treatment of vertigo and dizziness in clinical practice. This review aims to serve as a clinical companion for physicians of all specialties dealing with the primary diagnosis and treatment of vertigo and dizziness.

**Supplementary Information:**

The online version of this article (10.1007/s00106-025-01599-z) contains supplementary material, which is available to authorized users.

## Abstract (de)

**Hintergrund:**

Schwindel ist eines der häufigsten Leitsymptome in der klinischen Praxis, die Differenzialdiagnose stellt oftmals eine Herausforderung dar.

**Ziel der Arbeit:**

Die vorliegende Arbeit soll praxisorientierte und evidenzbasierte Empfehlungen zur Erstdiagnostik und -therapie von Schwindelsyndromen vermitteln.

**Material und Methoden:**

Der Konsensus einer interdisziplinären Arbeitsgruppe im Nachgang an eine Umfrage unter Schweizer Primärversorger:innen und Spezialist:innen (HNO, Neurologie) zu Diagnostik und Therapie bei Schwindel sowie die Ergebnisse einer Literaturrecherche in PubMed bis Oktober 2024 werden präsentiert.

**Ergebnisse und Schlussfolgerung:**

Eine strukturierte Anamnese und klinisch-neurootologische Untersuchung bilden die Grundlage für die Differenzialdiagnose der verschiedenen akuten (AVS) episodischen (EVS) und chronischen (CVS) vestibulären Syndrome (AVS: z. B. Schlaganfall oder akute unilaterale Vestibulopathie; EVS: z. B. benigner paroxysmaler Lagerungsschwindel [BPLS], M. Menière, vestibuläre Migräne, Vestibularisparoxysmie; CVS: z. B. bilaterale Vestibulopathie, „persistent postural perceptual dizziness“). Der vorliegende Artikel enthält Übersichten zu (i.) „red flags“ für eine potenziell gefährliche Ursache bei Patient:innen mit akutem Schwindel/Gangunsicherheit, (ii.) essenziellen klinisch-neurootologischen Untersuchungen bei Schwindel, (iii.) den Lagerungs- und Repositionsmanövern für BPLS der posterioren und lateralen Bogengänge und (iv.) den wichtigsten therapeutischen Ansätzen bei den o. g. vestibulären Syndromen sowie (v.) die „Top-10-Empfehlungen“ zu Anamnese, Diagnose und Therapie von Schwindelerkrankungen in der klinischen Praxis. Diese Zusammenstellung möge Ärzt:innen unterschiedlicher Disziplinen als tägliches „Vademecum“ bei der Erstdiagnose und -therapie von Schwindelerkrankungen dienen.

## Preliminary remarks

Following a published survey among Swiss primary care physicians and neuro-otology specialists (ear, nose, throat [ENT], neurology) on the diagnosis and treatment of vertigo and dizziness, it was decided to develop a guidance paper [1–4]. To this end, an interdisciplinary working group was formed including neurologists (authors AAT, HK, SD, RK), ENT specialists (CC, RP, RM, AWL, JD), a general practitioner (JB) from Switzerland and a representative from Germany (AZ). The work presented here reflects the consensus reached by this working group and should be considered a guidance paper for the primary diagnosis and treatment of patients with vertigo and dizziness in clinical practice.

## Frequency

Vertigo and dizziness are among the most frequent presenting symptoms in clinical practice, accounting for up to 8% of all medical consultations [5]. In about 10–15% of all patients, they are due to a serious but treatable disease [6]. A reliable causal relationship between the quality of symptoms (vertigo vs. dizziness vs. presyncope) and the underlying cause [7] is neither possible nor does it enable a distinction to be made between benign and dangerous diseases [8]. The broad spectrum of differential diagnoses, (too) short consultation time slots, and lack of expertise are important limiting factors among primary care physicians [1].

The aim of this article is to fill current gaps of knowledge and to provide pragmatic and evidence-based recommendations for the diagnosis and treatment of the dizzy patient.

## Diagnosis

### History taking

**Fig. 1:**
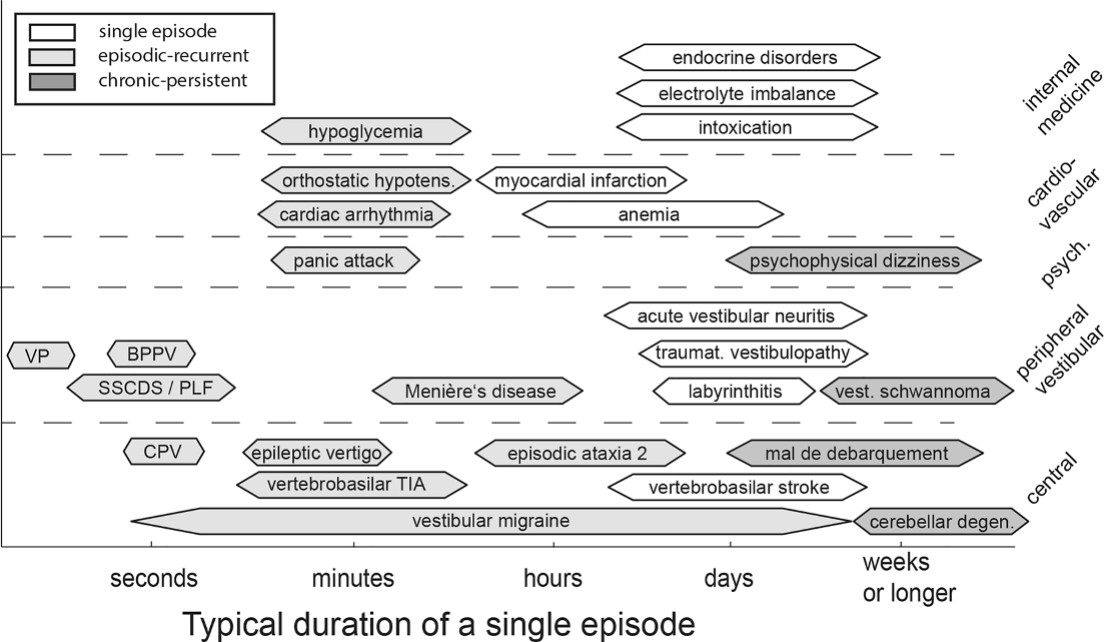
The most frequently encountered disorders associated with dizziness/vertigo and their typical ranges of symptom duration (modified after Büki and Tarnutzer [36], with permission for reuse from Oxford Publishing Limited). These include vestibular, central, psychiatric, internal medicine, and cardiovascular diseases. Single or recurrent dizzy spells are distinguished from chronic dizziness/vertigo. *BPPV* benign paroxysmal positional vertigo, *CPV* central positional vertigo, *PLF* perilymph fistula, *SSCDS* superior semi-circular canal dehiscence syndrome, *TIA* transient ischemic attack, *VP* vestibular paroxysmia

**Fig. 2:**
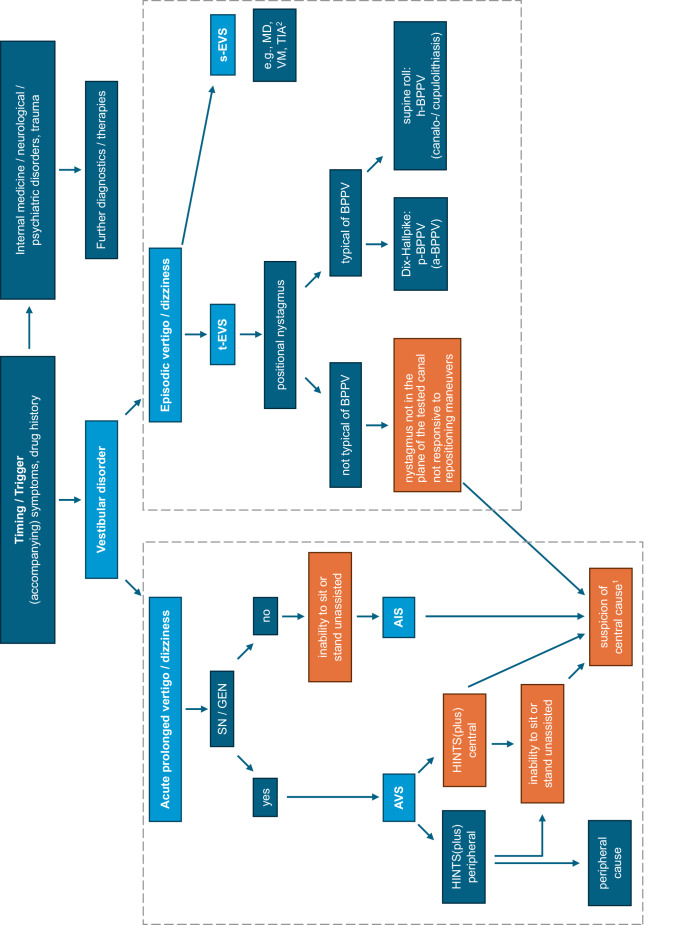
Differential diagnosis of vestibular disorders according to the “TiTrATE” (*Ti*ming, *Tr*iggers *A*nd *T*argeted *E*xaminations) approach. Boxes with orange background: “red flags” for a central vestibular disorder. ^1^Further red flags: purely vertical, purely torsional or vertical-torsional spontaneous nystagmus, ocular lateral deviation, saccadic pursuit, dysmetric saccades (see also Table 1). ^2^The distinction between an acute vestibular syndrome (AVS) and the first episode of a spontaneous episodic vestibular syndrome (s-EVS) is often possible only retrospectively. For details, see main text. *a* Anterior, *AIS* acute imbalance syndrome, *AVS* acute vestibular syndrome, *BPPV* benign paroxysmal positional vertigo, *EVS* episodic vestibular syndrome, *GEN* gaze-evoked nystagmus, *h* horizontal, *HINTS* Head Impulse, Nystagmus, Test of Skew, *MD* Menière’s disease, *p* posterior, *s* spontaneous, *t* triggered, *SN* spontaneous nystagmus, *TIA* transient ischemic attack, *VM* vestibular migraine

When taking the patient’s history, special emphasis should be laid on the duration of symptoms and frequency of episodes (acute and prolonged vs. episodic vs. chronic) as well as the presence/absence of triggers (so-called timing-and-triggers approach; [7]). While recurrent dizzy spells (episodic vestibular syndrome [EVS]) lasting minutes to hours may occur due to vestibular migraine or Menière’s disease, the differential diagnosis becomes much broader in the case of first episodes (including, e.g., a transient ischemic attack [TIA]). For acute and prolonged vertigo or dizziness (i.e., an acute vestibular syndrome [AVS]), the distinction between dangerous central (e.g., ischemic stroke) and benign peripheral (e.g., acute unilateral vestibulopathy [AUVP]) causes is essential (see Fig. 1). In addition, provoking factors (triggering the symptoms) have to be distinguished from exacerbating factors (worsening pre-existing symptoms). Accompanying symptoms, pre-existing disorders, and current medications are further important aspects in the history taking of dizzy patients. Figure 2 illustrates the diagnostic approach in the case of an AVS or EVS according to the “TiTrATE” (*Ti*ming, *Tr*iggers *A*nd *T*argeted *E*xaminations) scheme. The most important “red flags” are summarized in Table 1, and essential neuro-otologic terms are listed in the glossary (Supplementary material, Table S1).

**Table 1:** “Red flags” in patients with acute vertigo, dizziness, or gait imbalance

Red flag—vertigo/dizziness/gait imbalance plus …	Differential diagnosis	Recommended measure(s)
*History taking*		
Acute hearing loss without signs of an ear pathology including Menière’s disease (see also main text)	Central causes (mostly brainstem strokes in the AICA territory)	Referral to the nearest stroke unit for further diagnosis and treatment including brain MRI
Multiple vascular risk factors (ABCD2-score $\geq 4$)	Central, mostly ischemic causes	Referral to the nearest stroke unit for further diagnosis and treatment including brain MRI
Hyperacute onset without triggers	Central, mostly ischemic causes	Referral to the nearest stroke unit for further diagnosis and treatment including brain MRI
New-onset headache or neck pain ± new unilateral hearing loss	Increased intracranial pressure (e.g., due to a mass lesion in the posterior fossa or a sinus vein thrombosis), dissection of the vertebral artery	Referral to the emergency department for brain imaging (CT including post-contrast sequences and demonstration of sinus veins and arterial blood supply)
Transient loss of consciousness	Epileptic seizure, increased intracranial pressure with imminent herniation, subarachnoid hemorrhage, drug intoxication (e.g., due to neuroleptics, antiepileptic drugs), cardiac arrhythmia, basilar artery thrombosis	Referral to the emergency department for brain imaging (CT including CT angiography sufficient in most cases), EEG, ECG, therapeutic drug monitoring
Persistent vomiting, decreased food intake, reduced food absorption	Acute thiamine deficiency	Thiamine blood level measurement, immediate high-dose thiamine supplementation (3 × 200–500 mg/ day as a short infusion, for 5 days)
Fever	Meningitis, meningoencephalitis	Referral to the emergency department for brain imaging (non-contrast CT usually sufficient) and lumbar puncture
Diplopia, dysphagia, dysarthria, dysesthesia, dysmetria (“deadly Ds”)	Central causes in the brainstem/cerebellum (stroke, tumor, demyelinating lesion), drug intoxication	Referral to the nearest stroke unit for further diagnosis and treatment including brain MRI
*Bedside examination findings*		
Normal head impulse test (in the presence of spontaneous nystagmus or gaze-evoked nystagmus)	Central causes in the brainstem/cerebellum (stroke, tumor, demyelinating lesion), drug intoxication, first episode of a vestibular migraine	Referral to the nearest stroke unit for further diagnosis and treatment including brain MRI
Purely vertical, purely torsional, or torsional-vertical spontaneous nystagmus	Central causes in the brainstem/cerebellum (stroke, tumor, demyelinating lesion), drug intoxication, acute thiamine deficiency, first episode of a vestibular migraine	See above
Horizontal, persistent gaze-evoked nystagmus at eccentric gaze	Central causes in the brainstem/cerebellum (stroke, hemorrhage, tumor, demyelinating lesion), drug intoxication, acute thiamine deficiency, first episode of a vestibular migraine	See above
Clinically manifest vertical divergence (“skew deviation”) in the alternating cover test	Central causes in the brainstem/cerebellum (stroke, hemorrhage, tumor, demyelinating lesion)	See above
(New-onset) ocular motor deficits (saccadic pursuit, dysmetric saccades, rebound nystagmus, vertical nystagmus after horizontal head shaking)	Central causes in the brainstem/cerebellum (stroke, hemorrhage, tumor, demyelinating lesion, neurodegenerative)	In the case of acute symptom onset referral to the emergency department (including brain imaging), in the case of subacute/chronic onset referral for specialized neurological evaluation
Acute, new-onset unilateral hearing loss without signs of an ear pathology including Menière’s disease	Central causes (mostly brainstem strokes in the AICA territory)	Referral to the nearest stroke unit for further diagnosis and treatment including brain MRI
Severe truncal ataxia (inability to sit or stand unassisted)	Vertebrobasilar stroke, brainstem hemorrhage or cerebellar hemorrhage, demyelinating lesion	See above
Position-dependent nystagmus refractory to repeated repositioning maneuvers and/or with atypical presentation (beating direction does not match the beating direction of a given SCC)	Central causes in the brainstem/cerebellum (stroke, hemorrhage, tumor, demyelinating lesion), first episode of a vestibular migraine	See above
Abnormal vital signs (hypotonia, tachycardia, arrhythmia)	Internal medicine or cardiologic causes	Urgent cardiologic/internal medicine evaluation

*AICA* anterior inferior cerebellar artery, *CT* computed tomography, *ECG* electrocardiogram, *EEG* electroencephalography, *MRI* magnetic resonance imaging, *SCC* semicircular canal

#### Recommendation 1.

The search for acute dangerous causes of vertigo and dizziness should be prioritized, and special attention should be paid to accompanying neurological (e.g., double vision, [hemi-]ataxia, palsies and dysarthria), otological (otalgia, hearing loss), and cardiovascular (palpitations, arrhythmia, chest pain) symptoms [7].

#### Recommendation 2.

History taking should always include asking about the timing (frequency, acuity, duration of single episodes), triggers (provoking factors/exacerbating factors), accompanying symptoms, and the current medication.

#### Recommendation 3.

Overreliance on symptom quality should be avoided; thus, the type of dizziness should not lead to the exclusion of certain differential diagnoses.

### Clinical examination of the acutely dizzy patient

The distinction between benign and dangerous underlying disorders has priority [9]. If acute and prolonged vertigo or dizziness is accompanied by nausea/vomiting, gait imbalance, motion intolerance, and nystagmus, this is referred to as an “acute vestibular syndrome” (AVS). In cases with imbalance of stance and gait without accompanying nystagmus, it is considered an “acute imbalance syndrome” (AIS; Supplementary Table S1; Fig. 2).

In the clinical examination the search for subtle oculomotor signs and for imbalance of stance and gait is essential. This includes central oculomotor disorders without gaze palsies, such as spontaneous nystagmus (SN) beating in a vertical, torsional, or vertical-torsional plane; horizontal gaze-evoked nystagmus (GEN); ocular lateral deviation (OLD); saccadic pursuit; dysmetric saccades; and preserved angular vestibulo-ocular reflex (VOR; Table 1 and Supplementary Table S1; [10]). Obvious focal neurological findings are lacking in about half of all patients with central AVS. In this setting, the targeted neuro-otologic examination is more sensitive than the early (i.e., retrieved within the first 24–48 h of symptom onset) brain magnetic resonance imaging (MRI) including diffusion-weighted imaging sequences (with about 20% false-negative findings; [10]). The presence of an SN is indicative of an acute vestibular disorder (peripheral or central). An (isolated) vertical, isolated torsional, or a torsional-vertical SN each have a very high specificity for a central cause (Tables 1 and 2). By contrast, no distinction between a peripheral and a central cause is possible if a horizontal-torsional SN is observed [11].

**Table 2:** Essential bedside testing in the dizzy patient

Examination	Recommended for the following conditions/situations	How to interpret abnormal findings
Search for focal neurological findings	In all dizzy patients	Presence of focal neurological deficits → strongly suggestive of a central cause
Search for a spontaneous nystagmus with fixation preserved and denied (by use of Frenzel’s goggles)	In all dizzy patients	If horizontal nystagmus is seen, with torsion and/or reduction by fixation → more likely to be peripheral
		If purely vertical, purely torsional, or vertical-torsional and without fixation suppression → more likely to be central
Search for subtle oculomotor (and cochlear) signs (HINTS-plus)	In the case of acute prolonged vertigo or dizziness (AVS)	Head impulse test → Catch-up saccades to one side usually suggestive of a peripheral cause (ATTENTION: “false” abnormal HIT in 20% of patients with central AVS), normal HIT when presenting as AVS → suggestive of a central cause
		Eccentric gaze holding → Gaze-evoked nystagmus (i.e., drift of the eyes towards primary gaze and correcting saccade outwards) is highly suggestive of a central cause
		Alternating cover test → vertical deviation with catch-up saccade on refixation (“skew deviation”) is highly suggestive of a central cause
		New-onset unilateral hearing loss (evaluated by whispering words or finger rub) without signs of ear pathologies → suggestive of a central cause (“plus” criterion)
Search for truncal ataxia	In all dizzy patients	Unassisted sitting or standing without support not possible → highly suggestive of a central cause
		Standing without support (in tandem stance) and walking unassisted possible → peripheral or central
Provocation maneuvers for the posterior and lateral semicircular canals	In the case of typical provocation factors	Hallpike-Dix maneuver: torsional-geotropic nystagmus → BPPV of the ipsilateral posterior semicircular canal
		Supine roll maneuver: geotropic or apogeotropic purely horizontal nystagmus → BPPV of the lateral semicircular canal

*AVS* acute vestibular syndrome, *BPPV* benign paroxysmal positional vertigo, *HINTS* Head Impulse, Nystagmus, Test of Skew, *HIT* Head Impulse Test

### Performing the HINTS and evaluating stance and gait

Whenever patients present with acute prolonged vertigo or dizziness meeting diagnostic criteria for an AVS, i.e., they demonstrate either SN or GEN, performing the HINTS (*H*ead *I*mpulse, *N*ystagmus, *T*est of *S*kew) is essential (Fig. 2; Table 2, Supplementary Table S1). A reliable examination within a few minutes (duration 2–3 min) and an accurate interpretation of the findings are already possible after a few hours of dedicated training [12]. In this setting the HINTS enables the identification of central causes with high diagnostic accuracy (sensitivity: 95.3%; [10]). Thereby the distinction should be made between “central HINTS” and “peripheral HINTS.” The horizontal Head Impulse Test (HIT) assesses the integrity of the VOR of the horizontal semicircular canals (see Supplementary Table S1). High-velocity, small-amplitude (5–15° excursion) head rotations in an unpredictable direction and with varying amplitude should be applied while the patient is looking at the examiner’s nose [13]. Catch-up saccades are the hallmark sign of an impaired VOR on the side the head impulses were applied to. This finding is mandatory for AUVP, but it can also be observed in about 20% of all central causes, as the second neuron of the VOR is located within the brainstem vestibular nuclei. Therefore, only the combination of SN and a bilaterally preserved HIT confirms a central origin of the AVS. Other subtle oculomotor signs such as evaluating for eccentric gaze holding (“*N*ystagmus”: assessed at about 30° of gaze eccentricity, duration ≥10 s) and for vertical fixation during the alternating cover test (“*T*est of *S*kew”: one eye after the other is covered alternatingly with a frequency of about 0.5 Hz) should always be determined as well (Table 2; Fig. 2).

The HINTS are considered central if one or more of the signs are indicative of a central origin (Table 2 and Supplementary Table S1). In cases with peripheral HINTS, testing for a new-onset unilateral hearing loss should be added, which is referred to as “HINTS-plus” ([12]; Table 2, Supplementary Table S1, Fig. 2). This condition is highly suggestive of a central cause until proven otherwise (e.g., inner ear pathologies, such as the first episode of Menière’s disease). Otoscopy and a clinical hearing test should therefore be performed on all patients presenting with an AVS (see below).

Additionally (or alternatively—if the examiner has no experience in performing/interpreting the HINTS) a graded gait and truncal instability rating should be performed (Table 2; Fig. 2). This is especially important in cases with no SN or GEN but with ongoing imbalance of stance and gait. In such cases, an AIS is assumed. If patients are unable to stand or sit unassisted at all, this is highly suggestive of a central cause [12].

Because of acute treatment options available in central (mostly ischemic) causes of an AVS or AIS including intravenous thrombolysis (possible up to 9 h after symptom onset in selected cases [14]) and endovascular thrombectomy (possible up to 24 h after symptom onset in selected cases [15]), these patients should be preferentially evaluated in a hospital with a stroke unit.

### Clinical neurological examination in patients with spontaneous episodic or chronic dizziness/vertigo

In patients with *spontaneous* episodic or chronic dizziness/vertigo, a principal distinction should be made between peripheral vestibular, central vestibular, and non-vestibular causes (Fig. 1). Oculomotor signs (e.g., saccadic pursuit, dysmetric saccades, vertical SN) are indicative of a central origin (Tables 1 and 2); a bilaterally abnormal HIT in combination with a marked sway on the Romberg test speaks for a bilateral vestibulopathy. In those patients reporting vertigo or dizziness while standing or walking or demonstrating a chronic gait disorder, an extended spectrum of potential underlying causes including peripheral polyneuropathy, cerebellar ataxia, and extrapyramidal disorders should be considered.

### Positioning maneuvers

**Fig. 3:**
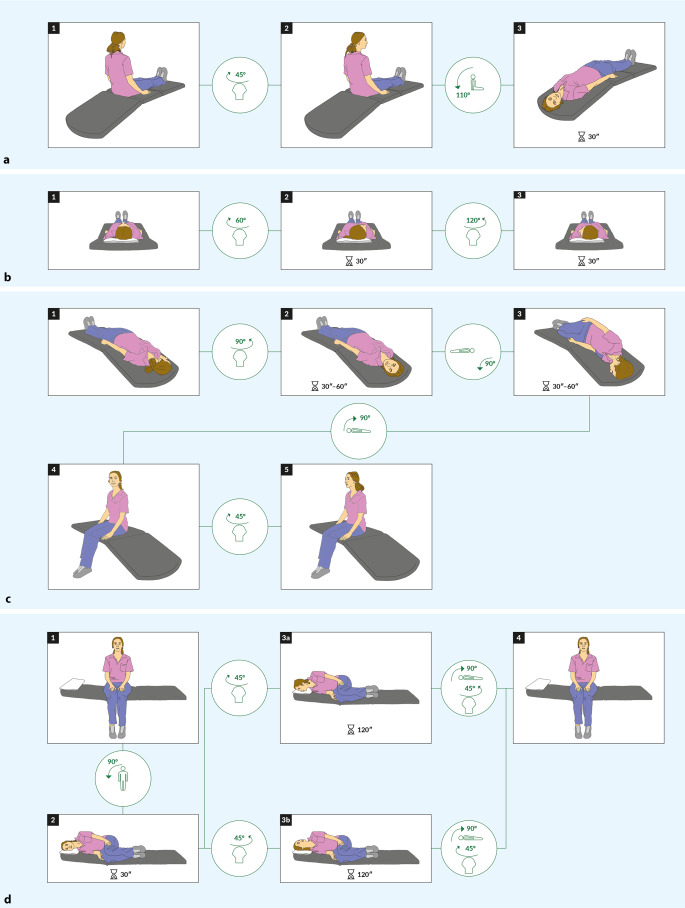
Provocation maneuvers and repositioning (liberation) maneuvers for diagnosing and treating benign paroxysmal positional vertigo (BPPV), illustrated stepwise and with pictograms indicating changes in head- and body position (note that the patient is always seen from behind). **a** Testing of the posterior semicircular canals (SCCs) is done by the Hallpike–Dix maneuver (illustrated here for the right posterior SCC). While sitting on the examination couch, the patient’s head is turned to either side by about 45°. Then the patient is brought to a head-hanging position (about 20° below the horizontal plane). Observation of a torsional-geotropic (i.e., rotational and beating towards the earth) nystagmus and reported vertigo or dizziness are highly suggestive of BPPV. **b** Testing of the lateral SCCs is done by the supine roll test. The patient is brought to a supine position (or with the upper part of the body elevated slightly by 10–20°). Then the patient’s head is turned to either side by about 60° and special attention is paid to the eventual occurrence of a horizontal geotropic (i.e., beating towards the earth) or apogeotropic (i.e., beating away from the earth) nystagmus (see main text for identification of the affected side). **c** The liberation maneuver for a right posterior canal BPPV by use of the Epley maneuver is done stepwise as indicated. Sufficiently long breaks (at least 30–60 s) per position should be applied. **d** In the case of lateral canal BPPV, the Gufoni liberation maneuver distinguishes between the geotropic variant (3a, shown for the left lateral SCC → a head rotation “nose down” is applied) and the apogeotropic variant (3b, shown for the right lateral SCC → a head rotation “nose up” is performed)

In cases with *triggered* episodic vertigo or dizziness, HINTS (plus) should not be performed in the first place, but positioning maneuvers should be applied instead to search for benign paroxysmal positional vertigo (BPPV; [16]; Table 2; Fig. 2). Due to the high incidence of BPPV, positioning maneuvers should be included in the clinical assessment of every dizzy patient. While the posterior semicircular canals (SCCs) are tested with the Hallpike–Dix maneuver (Fig. 3a), the supine roll maneuver is suitable for detecting BPPV of the lateral SCCs (Fig. 3b; Table 2). The presence of a positional nystagmus with a crescendo–decrescendo pattern and torsional-geotropic beating direction (i.e., beating towards the earth and rotational) in combination with reported transient vertigo or dizziness in the Hallpike–Dix position supports a diagnosis of BPPV in the ipsilateral posterior SCC. A latency of a few seconds and a duration of the observed nystagmus and the reported symptoms of 10–20 s are characteristic of canalolithiasis [16]. A horizontal geotropic (i.e., beating towards the earth) or apogeotropic (i.e., beating away from the earth) nystagmus accompanied by vertigo or dizziness during the supine roll maneuver is characteristic of a BPPV of the lateral SCC. While lying on the affected side produces more nystagmus for the geotropic variant, it is the side with less nystagmus that indicates the affected side in cases with apogeotropic nystagmus. The presence of geotropic (or apogeotropic) nystagmus when turning the head to the left side *and* to the right side is mandatory for the diagnosis of lateral canal BPPV. Computed tomography or MRI of the brain is not routinely required [17]. However, brain MRI should be considered for treatment-refractory or atypical cases (Table 1; Fig. 2). In cases of a persistent positional nystagmus (i.e., lasting > 60 s), otoconia are likely to be attached to the cupula, indicative of cupulolithiasis.

### Ear examination

An otoscopy is mandatory in every dizzy patient. The search for signs of inflammation (e.g., otorrhea, redness/bulging of the ear drum, vesicles/crusts), trauma (e.g., fractures with visible step-offs/blood in the ear canal, ear drum perforation), and masses (e.g., squamous debris/epitympanal retraction pocket of the ear drum in the case of cholesteatoma) has priority. In the case of reported hearing loss, a preliminary hearing test with a tuning fork (440 Hz; Rinne and Weber test) should be performed in order to distinguish between conductive hearing loss (Rinne negative on the affected side, Weber lateralizes to the affected side) and sensorineural hearing loss (Rinne positive, Weber lateralizes to the healthy side). Patients with abnormal findings on otoscopy and/or with new-onset hearing loss should be further evaluated by an ENT specialist.

#### Recommendation 4.

The HINTS (plus) has a high diagnostic accuracy for distinguishing between peripheral and central causes *only* in patients with AVS (i.e., presenting with SN and/or GEN and acute-onset, persistent vertigo or dizziness). In the absence of any nystagmus (i.e., presenting as an AIS), a graded gait and truncal instability rating should be performed preferentially.

#### Recommendation 5.

Positioning maneuvers are especially (but not solely) recommended in patients with intermittent, position-dependent vertigo or dizziness. Testing should always include both the posterior and the lateral canals.

#### Recommendation 6.

All dizzy patients should undergo a neurological examination, otoscopy, and preliminary testing for hearing loss.

## Treatment principles

For the treatment of vertigo and dizziness, pharmacologic, physiotherapeutic, psychotherapeutic/behavioral and—much less frequently—surgical approaches may be considered. Often several treatment modalities are combined. Whenever possible, treatment strategies addressing the pathophysiology and the underlying cause are selected. Preferred treatment strategies for the most important disorders leading to vertigo and dizziness are summarized in Table 3. Importantly, evidence for many drug treatment strategies considered for the dizzy patient is low and drug use is off-label. During the consultation with the dizzy patient, healthcare providers should address the prognosis of the disease, disclose off-label use of medication, and promote realistic expectations regarding delay in response to an intervention.

**Table 3:** Treatment strategies for the most common neuro-otologic causes of vertigo/dizziness and gait imbalance

	Drug treatment		Other (non-pharmaceutical) treatment options			
*Diagnosis*	*Acute phase/symptomatic*	*Prophylaxis*	*Acute phase/symptomatic*	*Prophylaxis/* *additional therapeutic options*	*Follow-up*	*Recommendations for further diagnostic work-up/treatment*
Benign paroxysmal positional vertigo (BPPV)	Antiemetics and/or sedatives for premedication before repositioning maneuvers if needed	Vitamin D_3_ supplementation recommended (in the case of recurrent BPPV) [26]	Repositioning maneuvers (physician’s office/emergency department, at home, Fig. 3, S1–2), physical therapy (including home exercises)	Instructions for self-repositioning maneuvers by use of hand-outs, videos (e.g., https://novel.utah.edu), smartphone apps	After 3–7 days (phone call, remotely by video, in the physician’s office)	Referral to specialist (neurologist, ENT), quantitative vestibular testing and brain MRI in the case of treatment-refractory complaints
Acute unilateral vestibulopathy	Consider steroids (taking into account comorbidities) according to S2k guideline “Vestibuläre Funktionsstörungen” (DGHNO-KHC/DGN) [27] May be considered: antiemetics (domperidone, metoclopramide, ondansetron) and/or antivertiginous drugs (cinnarizine ± dimenhydrinate) for a max. of 2–3 days*, Ginkgo biloba *extract (EGb 761) to support central adaptation [28, 29]	None	Physical therapy (vestibular rehabilitation therapy, VRT) [30]	Instructions for VRT at home by use of hand-outs, videos, smartphone apps [31]	Follow-up after 2–4 weeks recommended	Urgent referral to the emergency department or the specialist (neurology, ENT) in severe cases or suspected central causes and for detailed qualitative/quantitative vestibular testing (see Table 1 “red flags”)
Ischemic/hemorrhagic stroke	Consider antiemetics (domperidone, metoclopramide, ondansetron) for patient transfer	Secondary prophylaxis according to guidelines (DGN [32], AHA/ASA [33])	Neuro-rehabilitation (outpatient, in-house)	Lifestyle modifications (according to guidelines for secondary prophylaxis [33]) Additional measures, see S2k-guideline (DGN), AHA/ASA guideline [33]	Routine follow-up according to specialist	Immediate referral to closest stroke unit
Vestibular migraine (VM)	Acute drug treatment according to guidelines (see migraine attack treatment recommendations of the Swiss Headache Society, www.headache.ch and DGN [34])	Decision for prophylactic treatment together with specialist recommended (see migraine prophylactic treatment, www.headache.ch and DGN [34])	None	Lifestyle modifications Additional measures, see guidelines DGN and Swiss Headache Society	Follow-up consultation recommended 10–12 weeks after starting prophylactic treatment	Referral to neuro-otology specialist/to a specialized dizzy clinic recommended; consider brain MRI for search of secondary causes of dizziness/headache
Menière’s disease	Antiemetic drugs (domperidone, metoclopramide, ondansetron)	Decision for prophylactic treatment together with specialist recommended (for details, see [27, 35])	None	Individual multimodal treatment concept, “shared decision-making” including lifestyle modifications, auditory rehabilitation, physical therapy, psychotherapy, self-help groups (for details, see [27, 35])	Usually with neuro-otology specialist	Referral to neuro-otology specialist/to a specialized dizzy clinic recommended; exclude structural causes (brain MRI), consider hydrops MRI
Vestibular paroxysmia	None (attacks usually too short for symptomatic treatment)	Decision for prophylactic treatment together with specialist recommended (carbamazepine, oxcarbazepine, lacosamide off-label use) [27]	None	None	Usually with neuro-otology specialist	Referral to neuro-otology specialist/to a specialized dizzy clinic recommended; exclude structural causes (brain MRI)
Persistent postural-perceptual dizziness/functional dizziness	Use of antiemetics or antivertiginous drugs not recommended	Decision for prophylactic treatment together with specialist recommended (usually antidepressants)	None	Usually multimodal treatment approach including patient education (vestibular.org), behavioral therapy and physical therapy (including home exercises) [25]	Usually with neuro-otology specialist	Referral to neuro-otology specialist/to a specialized dizzy clinic recommended; treatment of the condition that triggered the complaints (e.g., BPPV, VM)
Bilateral vestibulopathy	None	None	None	Physical therapy (including exercises at home, hand-outs, smartphone apps) Avoid ototoxic drugs Treatment of other disorders affecting the sensory organs (e.g., vision, hearing)	Follow-up with neuro-otology specialist after 6 months recommended	Referral to neuro-otology specialist/to a specialized dizzy clinic recommended; search for other causes (e.g., CANVAS)

*AHA* American Heart Association, *ASA* American Stroke Association, *CANVAS* cerebellar ataxia neuropathy vestibular areflexia syndrome, *MRI* magnetic resonance imaging, *DGHNO-KHC* Deutsche Gesellschaft für Hals-Nasen-Ohren-Heilkunde, Kopf- und Halschirurgie (German Society for Otorhinolaryngology, Head and Neck Surgery), *DGN* Deutsche Gesellschaft für Neurologie (German Society for Neurology), *VRT* vestibular rehabilitation therapy

## The most important vestibular disorders

### Acute vestibular syndrome/acute imbalance syndrome

In the case of AVS/AIS, the search for an ischemic vertebrobasilar stroke needs to be prioritized, as this is the single most frequent central cause for central AVS (80% of cases; see Tables 1–3 and Fig. 2). Other important central AVS causes include drug intoxications (e.g., due to neuroleptics or antiepileptic drugs), a first episode of vestibular migraine, or thiamine deficiency. On the other hand, AUVP (“vestibular neuritis”) is the most frequent cause of a peripheral AVS. If HINTS (plus) is “peripheral” in the AVS patient, available acute treatment options (see Table 3) should be considered. Importantly, intake of antivertiginous drugs and antiemetics should be limited to a maximum of 2–3 days in order to avoid inhibiting central compensation.

### Episodic vestibular syndrome

In EVS patients the distinction between triggered and spontaneous occurrence is key (see Fig. 2). Whenever positioning maneuvers in patients with triggered EVS (t-EVS) demonstrate a nystagmus pattern consistent with BPPV, suitable liberation maneuvers should be applied (see Fig. 3). If no nystagmus can be provoked or in cases with dizziness emerging only when sitting up, the search for orthostatic hypotension should be initiated. However, negative positioning maneuvers with a history of t‑EVS do not exclude a recent BPPV that has already resolved spontaneously.

For the treatment of posterior canal BPPV (representing about 80% of all cases), the Epley maneuver (Fig. 3c) or the Semont-plus maneuver (see Supplementary Fig. S1) should be considered [16, 18]. In the case of a lateral canal BPPV (as seen in about 20% of cases), a Gufoni maneuver should be applied preferentially (see Fig. 3d), alternatively a 360° barbecue maneuver in the direction to the non-affected ear with subsequent resting on the non-affected ear (“roll and rest”) may be considered (see Supplementary Fig. S2; [19]). For the geotropic variant, the Gufoni maneuver is applied “nose down” to the healthy side. In the case of the apogeotropic variant, it is performed “nose up” to the affected side. For the Gufoni maneuver, repositioning is always towards the side with less nystagmus. If no distinction can be made between the side with more nystagmus and the side with less nystagmus in the supine roll test, the patient should be asked about the intensity of vertigo in both positions and the Gufoni liberation maneuver should be made to the side with less vertigo.

After the liberation maneuver, the provocation maneuver should be repeated to assess treatment response (and a repeated liberation maneuver should be applied in those cases with persistent symptoms). Self-repositioning maneuvers are recommended in patients with recurrent BPPV. A follow-up visit (remotely or in-person) is suggested [20]. Patients with BPPV should be informed that residual complaints (gait imbalance, drowsiness) may be present for a few days after successful liberation maneuvers. Furthermore, the risk for recurrent BPPV of about 15–20% over a period of 12 months should be disclosed to the patient. Whenever provocation and/or liberation maneuvers in suspected BPPV cannot be performed on the bench — as, e.g., in patients with reduced mobility, spinal cord disease, muscle palsies or extrapyramidal tract syndrome — use of a rotatory chair such as the TRV chair (Interacoustics, Denmark or the Rotundum, Balcare, Switzerland) is recommended. Importantly, red flags in patients with suspected BPPV should be remembered, including repeatedly unsuccessful liberation maneuvers, atypical nystagmus patterns, or the presence of focal neurologic abnormalities (see Table 1 and Fig. 2).

In patients with spontaneous EVS (s-EVS), the distinction between vestibular migraine (VM) and Menière’s disease has priority (see Fig. 2). While a positive ongoing or past history of migraine headaches is mandatory for a diagnosis of VM, documented sensorineural hearing loss affecting the low frequencies on pure tone audiometry is required to make a diagnosis of Menière’s disease according to the diagnostic criteria of the ICVD (International Classification of Vestibular Disorders). The ICVD diagnostic criteria for VM require at least five episodes of vertigo or dizziness of moderate or high intensity (lasting between 5 min and 72 h) with accompanying migraine-associated symptoms in at least every other attack and a diagnosis of migraine ([21]; for therapeutic options see, [22] and Table 3). For a diagnosis of Menière’s disease, at least two episodes with vestibular and cochlear symptoms (lasting between 20 min and 12 h) are required [23]. Selected treatment options for Menière’s disease are referred to in Table 3 and should be individually adjusted by ENT specialists.

### Chronic vestibular syndrome

In patients with persistent vertigo or dizziness, provoking and aggravating factors need to be identified. If vertigo and/or gait imbalance occurs only while standing or walking, peripheral polyneuropathy, chronic unilateral vestibular loss, bilateral vestibulopathy, and central (neurodegenerative, chronic microvascular) causes should be considered. Reported blurred vision during fast head turns favors a diagnosis of bilateral vestibulopathy. Likewise, the presence of a bilaterally abnormal head impulse test, a tendency to fall during the Romberg test on foam, a decrease in visual acuity of $\geq$2 lines when applied during head shaking (so-called dynamic visual acuity) suggest bilateral vestibulopathy (see [24]). If no peripheral vestibular deficit can be identified in CVS patients, central and functional disorders should be considered as well. In the case of central signs, such as cerebellar ataxia or hypokinesia, a specialized neurological assessment should always be ordered.

In patients presenting with persistent dizziness for $\geq$3 months with exacerbation when upright, during passive or active motion, or when exposed to moving visual stimuli or complex visual patterns leading to a significant impact on activities of daily life, functional dizziness (persistent postural-perceptual dizziness) should be considered. A history of acute vertigo or psychosocial stress is the most frequently identified trigger [25]. Treatment of persistent postural-perceptual dizziness is usually multimodal (see Table 3).

#### Recommendation 7.

In the case of BPPV, liberation maneuvers (Semont-plus/Epley for posterior canals; Gufoni/barbecue for lateral canals) should be performed.

#### Recommendation 8.

If a stroke is suspected, immediate referral to the closest stroke unit is important, whereas in the case of suspected acute thiamine deficiency, high-dose vitamin B_1_ supplementation has priority.

#### Recommendation 9.

Antivertiginous drugs and antiemetics should be taken no longer than 2–3 days in a row.

#### Recommendation 10.

Treatment of vestibular migraine, Menière’s disease, and functional dizziness should be offered in close collaboration with a neuro-otology specialist.

## Outlook

In the face of current knowledge gaps of primary care physicians in the care of dizzy patients [1], intensified education and training are essential to reduce the rate of unclear cases, referral to specialists, and unnecessary diagnostic testing and to improve prognosis at the same time. Primary care providers should be taught how to apply the HINTS in AVS and the provocation/liberation maneuvers in cases of suspected BPPV [2].

## Practical conclusion

- In the dizzy patient, a structured approach including targeted history taking (including duration and frequency of attacks and provocation factors) and a focused neuro-otologic examination including the search for subtle oculomotor signs are essential (“TiTrATE” approach).
- Identifying dangerous underlying causes has priority. In the case of acute vestibular syndrome or acute imbalance syndrome, these include vertebrobasilar stroke, whereas for episodic vestibular syndromes, cardiac arrhythmia and transient ischemic attacks need to be considered.
- Primary care physicians should be trained in performing HINTS and provocation/liberation maneuvers (especially for the lateral canals).
- Drug treatments for dizziness should be evidence-based whenever possible and should be periodically re-evaluated regarding treatment response and side effects.

## Supplementary Information

Tab. S1: key terms—definitions; Fig. S1: the Semont-plus maneuver for treating posterior-canal BPPV; Fig. S2: The 360° barbecue maneuver (“Lempert maneuver”) for treating a lateral-canal BPPV (shown here for the right-lateral canal [geotropic variant] with sitting-up at the end)

## Abbreviations

AIS: Acute imbalance syndrome
AUVP: Acute unilateral vestibulopathy
AVS: Acute vestibular syndrome
BPPV: Benign paroxysmal positional vertigo
CT: Computed tomography
CVS: Chronic vestibular syndrome
EVS: Episodic vestibular syndrome
GEN: Gaze-evoked nystagmus
HINTS: Head Impulse, Nystagmus, Test of Skew
HIT: Head impulse test
ICVD: International Classification of Vestibular Disorders
MRI: Magnetic resonance imaging
PPPD: Persistent postural perceptual dizziness
SCC: Semicircular canal
SN: Spontaneous nystagmus
TIA: Transient ischemic attack
TiTrATE: Timing, Triggers And Targeted Examinations
VM: Vestibular migraine
VOR: Vestibulo-ocular reflex

## Declarations

This article does not contain any studies with human participants or animals performed by any of the authors.

The supplement containing this article is not sponsored by industry.
