# Erratum to: Diagnosis and treatment of vertigo and dizziness

**DOI:** 10.1007/s00106-025-01670-9

**Published:** 2025-09-26

**Authors:** Alexander Andrea Tarnutzer, Hassen Kerkeni, Suzie Diener, Roger Kalla, Claudia Candreia, Renato Piantanida, Raphaël Maire, Antje Welge-Lüssen, Joris Budweg, Andreas Zwergal, Julia Dlugaiczyk

**Affiliations:** 1 Neurology, Cantonal Hospital of Baden, Baden, Switzerland; 2 University of Zurich (UZH), Zurich, Switzerland; 3 Department of Neurology, Bern University Hospital, University of Bern, Bern, Switzerland; 4 Practice Neurology, St. Gallen, Switzerland; 5 Department of Otorhinolaryngology, Head and Neck Surgery, Cantonal Hospital Lucerne, Lucerne, Switzerland; 6 Department of Otolaryngology-Head and Neck Surgery, Ospedale Regionale Di Lugano, EOC, Lugano, Switzerland; 7 Department of Otorhinolaryngology/Head & Neck Surgery, Lausanne University Hospital, Lausanne, Switzerland; 8 Department of Otorhinolaryngology, Head and Neck Surgery, Basel University Hospital, University of Basel, Basel, Switzerland; 9 Hausarztzentrum Bethesda, Basel, Switzerland; 10 Department of Neurology, LMU University Hospital, Munich, Germany; 11 German Center for Vertigo and Balance Disorders (DSGZ), LMU University Hospital, Munich, Germany; 12 Department of Otorhinolaryngology, Head and Neck Surgery, University Hospital Zurich (USZ), Raemistrasse 100, 8091 Zurich, Switzerland; 13 Interdisciplinary Center for Vertigo, Balance and Ocular Motor Disorders, University Hospital Zurich, Zurich, Switzerland

**Erratum to:**

**HNO 2025**

10.1007/s00106-025-01599-z

In Table 3 of this article, entries in the second column/heading “Drug treatment/Acute phase/symptomatic” were incorrect. The text was incorrecty given as “May be considered: antiemetics (domperidone, metoclopramide, ondansetron) and/or antivertiginous drugs (cinnarizine ± dimenhydrinate) for a max. of 2–3 days, Ginkgo biloba extract (EGb 716) to support central adaptation [28, 29]” but should have been “May be considered: antiemetics (domperidone, metoclopramide, ondansetron) and/or antivertiginous drugs (cinnarizine ±dimenhydrinate) for a max. of 2–3 days, Ginkgo biloba extract (EGb 761) to support central adaptation [28, 29]”.

Supplementary file: In Table S1, Term “Acute imbalance syndrome (AIS)” the Definition should have read “… without spontaneous or gaze-evoked nystagmus” instead of “… with spontaneous or gaze-evoked nystagmus”.

The original article has been corrected.

## Supplementary Information

Tab. S1: key terms—definitions; Fig. S1: the Semont-plus maneuver for treating posterior-canal BPPV; Fig. S2: The 360° barbecue maneuver (“Lempert maneuver”) for treating a lateral-canal BPPV (shown here for the right-lateral canal [geotropic variant] with sitting-up at the end)

