# Supplementary material for: Diagnosis and treatment of vertigo and dizziness: Interdisciplinary guidance paper for clinical practice
Source: HNO. 2025 Jun 17;73(Suppl 3):357–69. doi: 10.1007/s00106-025-01599-z (PMC12672759; doi:10.1007/s00106-025-01599-z)
Supplement: Supplementary file 1 — Tab. S1: key terms—definitions; Fig. S1: the Semont-plus maneuver for treating posterior-canal BPPV; Fig. S2: The 360° barbecue maneuver (“Lempert maneuver”) for treating a lateral-canal BPPV (shown here for the right-lateral canal [geotropic variant] with sitting-up at the end) [file 106_2025_1599_MOESM1_ESM.pdf]

Supplementary files

Table S1: key terms – definitions (1, 2)

**Term**	**Definition**
**Symptoms**	
Dizziness	Dizziness is the sensation of disturbed or impaired spatial orientation without a false or distorted sense of motion
Vertigo	Vertigo is the sensation of self-motion when no self-motion is occurring or the sensation of distorted self-motion during an otherwise normal head movement
**Vestibular syndromes**	
Acute vestibular syndrome (AVS)	Acute prolonged (lasting >24h) vertigo/dizziness with accompanying nausea/vomiting, gait imbalance, motion intolerance and (non-mandatory) a spontaneous or gaze-evoked nystagmus.
Acute imbalance syndrome (AIS)	Acute prolonged imbalance of stance and gait (probably accompanied by vertigo/dizziness) without spontaneous or gaze-evoked nystagmus.
Episodic vestibular syndrome (EVS)	Recurrent, intermittent vertigo or dizziness lasting seconds, minutes, hours or days with symptom-free intervals between episodes. Occurrence is either spontaneous or triggered (e.g. by positional changes)
Chronic vestibular syndrome (CVS)	Chronic (persistent) vertigo or dizziness
**Clinical findings**	
Spontaneous nystagmus	Involuntary rhythmic eye movements in primary gaze (straight-ahead). Referred to as a jerk nystagmus if a slow phase and a fast phase can be distinguished. The direction of the fast phase defines the beating direction of the nystagmus.
Gaze-evoked nystagmus	Nystagmus that changes its beating direction depending on direction of gaze and only occurs at eccentric gaze (horizontal or vertical). Due to an inability to keep gaze stable at an eccentric position, the eyes are drifting back to primary gaze (slow phase, centripetal), that is followed by a compensatory (fast phase) outward (centrifugal).
Skew deviation	Vertical ocular misalignment due to lesions along the peripheral or central vestibular pathways, assessed by alternatively covering und uncovering on eye (Test of Skew).
Vestibulo-ocular reflex (VOR)	This reflex arc forwards sensory input signals from the semicircular canals in the inner ear through the vestibular nerve to the brainstem vestibular nuclei and eventually to the ocular motor nuclei and the eye muscles. It immediately compensates for head turns by eye rotations into the opposite direction, allowing the steady fixation of an object in space during head motion. The head-impulse test is used to examine this reflex arc.
HINTS (3)	Bedside examination of subtle oculomotor signs (integrity of the horizonal VOR (**H**ead-**I**mpulse), eccentric gaze holding (**N**ystagmus), vertical divergence in the alternating cover test (**T**est-of-**S**kew)) in the AVS patient. HINTS are considered «central» if one or several of the following signs are present: normal head-impulse test, horizontal gaze-evoked nystagmus, skew deviation. HINTS are defined as «peripheral» if a unilaterally abnormal head-impulse test is found and no gaze-evoked nystagmus or Skew deviation is observed.

HINTS plus (4)	Examination of HINTS (see above) plus additional search for a new-onset unilateral hearing loss in the patient with peripheral HINTS. The HINTS plus are considered as «central», is a new-onset unilateral hearing loss is demonstrated (by use of whispering words, tuning forks, finger rub).

Figure S1 – the Semont-plus maneuver for treating posterior-canal BPPV

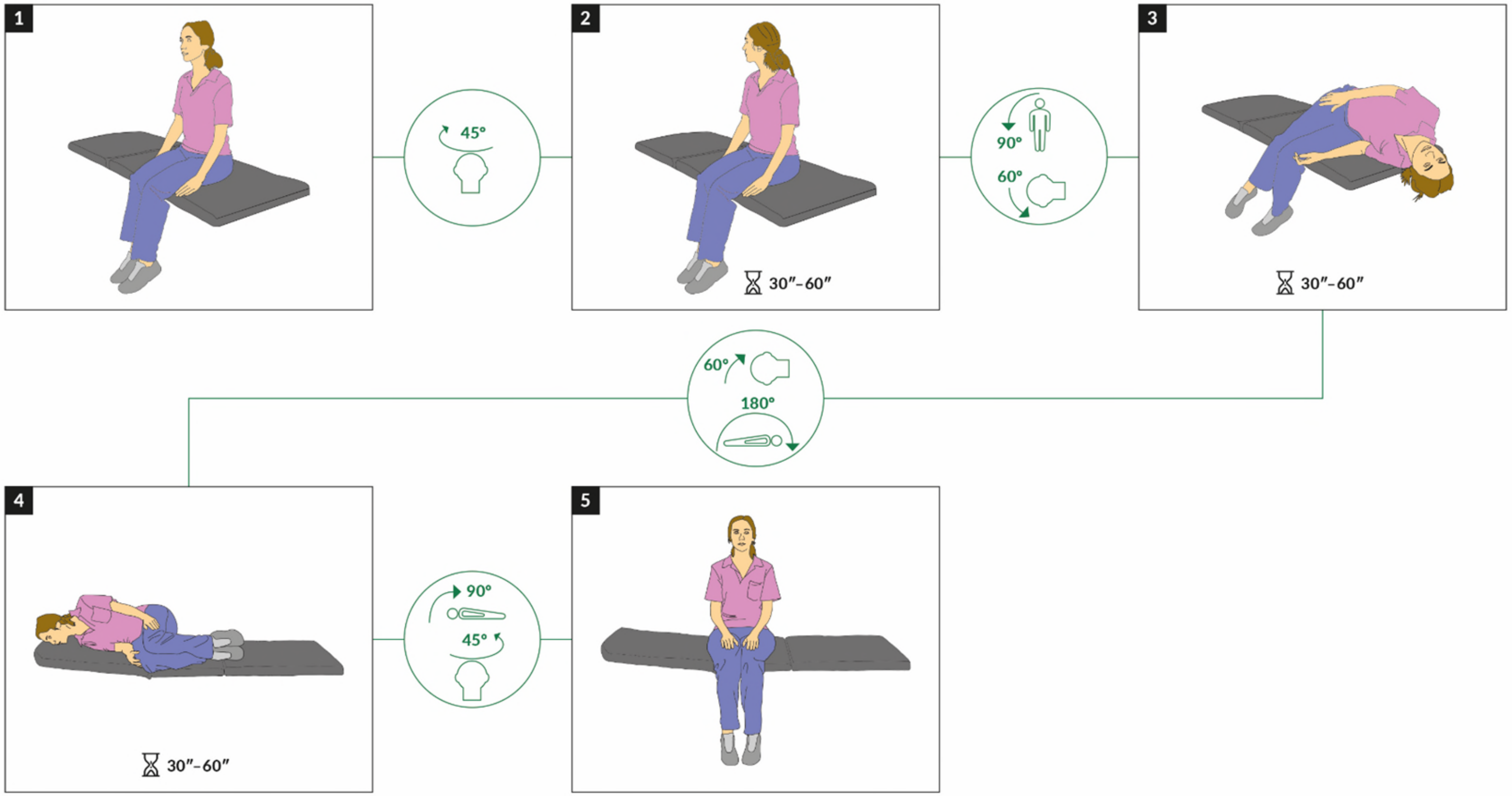

Figure S2 – The 360° Barbecue maneuver («Lempert maneuver») for treating a lateral-canal BPPV (shown hear for the right-lateral canal (geotropic variant) with sitting-up at the end)

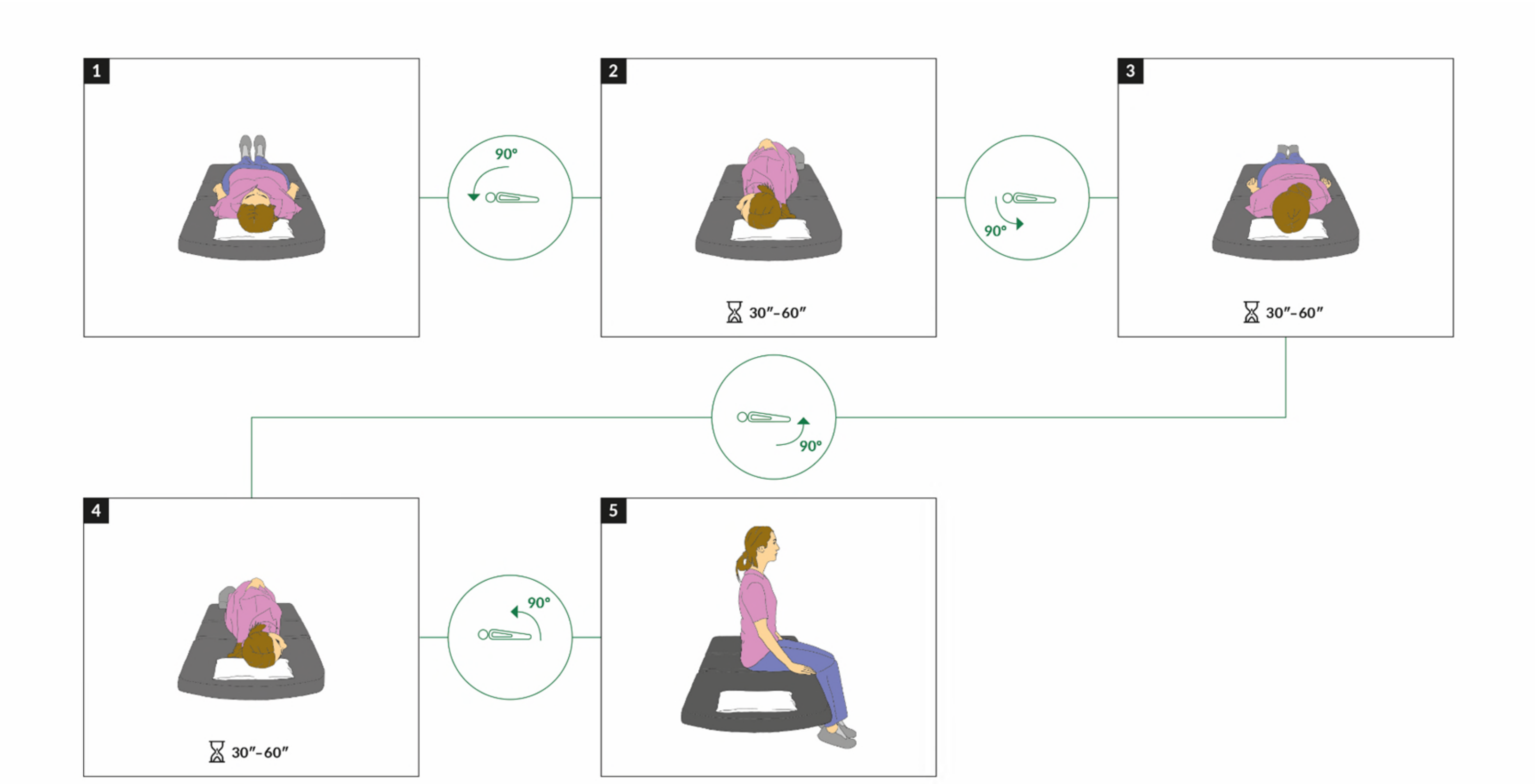

Figure legend: for the geotropic variant repositioning is towards the side with less nystagmus, for the apogeotropic variant (not shown here) repositioning is towards the side with more nystagmus.

References

1. Newman-Toker DE, Edlow JA. TiTrATE: A Novel, Evidence-Based Approach to Diagnosing Acute Dizziness and Vertigo. Neurol Clin. 2015;33(3):577-99, viii.
2. Bisdorff AR, Staab JP, Newman-Toker DE. Overview of the International Classification of Vestibular Disorders. Neurol Clin. 2015;33(3):541-50, vii.
3. Kattah JC, Talkad AV, Wang DZ, Hsieh YH, Newman-Toker DE. HINTS to diagnose stroke in the acute vestibular syndrome: three-step bedside oculomotor examination more sensitive than early MRI diffusion-weighted imaging. Stroke. 2009;40(11):3504-10.
4. Newman-Toker DE, Kerber KA, Hsieh YH, Pula JH, Omron R, Saber Tehrani AS, et al. HINTS outperforms ABCD2 to screen for stroke in acute continuous vertigo and dizziness. Acad Emerg Med. 2013;20(10):986-96.
